# Leitlinien zur molekulargenetischen Diagnostik: Fragiles-X-Syndrom und andere FMR1-assoziierte Syndrome

**DOI:** 10.1515/medgen-2021-2059

**Published:** 2021-05-14

**Authors:** Dieter Gläser, Deutsche Gesellschaft für Humangenetik e.V., Berufsverband Deutscher Humangenetiker e.V., Katrin Hinderhofer

**Affiliations:** Genetikum, Wegenerstr. 15, D-89231Neu-Ulm, Deutschland; Universitätsklinikum Heidelberg, Institut für Humangenetik, Im Neuenheimer Feld 366, D-69120Heidelberg, Deutschland

Das Fragile-X Syndrom (FXS, OMIM 300624), u. a. auch nach seinen Erstbeschreibern als Martin-Bell Syndrom bekannt, ist eine *neuronale Entwicklungsstörung*. Diese Form einer X-chromosomalen geistigen Behinderung tritt bei etwa einem unter 4000 Jungen und etwa einem unter 6000 Mädchen auf. Das Fragile-X assoziierte Tremor/Ataxie Syndrom (FXTAS, OMIM 300623) indessen ist eine *neurodegenerative Erkrankung* bei Vorliegen einer Prämutation. Die Fragile-X assoziierte prämature Ovarialinsuffizienz (FXPOI, OMIM 311360) beschreibt eine Störung in den Ovarien. Etwa 2 % der Frauen mit prämaturer Ovarialinsuffizienz haben eine FMR1-Prämutation. Diese drei FMR1-assoziierten Syndrome werden durch Veränderungen in dem Gen *FMR1* (OMIM 309550) verursacht, haben aber unterschiedliche pathophysiologische Ursachen.

## Einleitung und Definitionen

Die Bezeichnung „**Fragiles-X**“ leitet sich ab von einer spezifischen *chromosomalen* fragilen Stelle (FRAXA), an der das X-Chromosom zu mikroskopisch erkennbaren Chromatidveränderungen neigt, wenn Zellen von Betroffenen *ex vivo* unter bestimmten Bedingungen vermehrt werden. Die zugrundeliegende molekulare Veränderung betrifft das X-chromosomale Gen ***FMR1***(*fragile X mental retardation 1*). Dieses enthält in der nichttranslatierten Region (5’UTR) des ersten Exons einen variablen repetitiven Abschnitt mit tandemartig wiederholten CGG-Tripletts, der als **CGG-Repeat** bezeichnet wird. Unauffällige *FMR1*-Allele enthalten in unserer Population häufig 29 oder 30 Tripletts. Die Abfolge reiner CGGs ist typischerweise an etwa jeder 10. Position durch ein AGG unterbrochen.

### Fragiles-X-Syndrom (FXS)

Das FXS ist die häufigste monogen vererbte Form geistiger Behinderung. Die charakteristischen Symptome des FXS sind kognitive Entwicklungsstörungen sowie somatische Auffälligkeiten (große Ohren, langes schmales Gesicht, vorspringende Stirn, vorspringendes eckiges Kinn, hoher Gaumen, postpubertäre Makroorchidie, Mitralklappenprolaps, Sehstörungen) ferner Entwicklungs- und Verhaltensmerkmale (verzögerte Sprachentwicklung, Impulsivität, Hyperaktivität, soziale Scheu, Aufmerksamkeitsdefizit, autistoide Zeichen, Krampfanfälle, Hypersensitivität, schnelles repetitives Sprechen, Perseveration, Schlafstörungen). Die Bandbreite des kognitiven Entwicklungsrückstandes reicht von Lernschwierigkeiten bis zu schwergradiger geistiger Behinderung. Jungen sind generell stärker beeinträchtigt als Mädchen, wobei unter den Mädchen nur eine Minderheit keine klinischen Auffälligkeiten zeigen.

### Fragile-X assoziierte Tremor/Ataxie Syndrom (FXTAS)

Das FXTAS ist eine spätmanifestierende neurodegenerative Störung bei Trägern eines prämutierten *FMR1*-Gens. Bei *FMR1*-Allelen im Prämutationsbereich ist die Transkriptionsrate häufig mehrfach erhöht. Die pathophysiologische Ursache des neuronalen Zelluntergangs ist wahrscheinlich auf eine Toxizität der überexprimierten *FMR1*-mRNA zurückzuführen. Die zelluläre RNA-Menge steigt mit zunehmender Länge des prämutierten Repeats und fällt zum Ende des Prämutationsbereiches hin wieder ab. Bei vollmutierten CGG-Repeats (>200 Tripletts), die ausnahmsweise unmethyliert und transkriptionsaktiv bleiben, ist die Transkriptionsrate in der Regel nicht hoch genug, um neurotoxisch zu wirken.

Etwa 80 % aller Betroffenen haben einen Intensionstremor und/oder eine Gangataxie, meistens jedoch beide Symptome [1]. Weitere mögliche Krankheitsmerkmale sind vorzeitige Demenz, psychiatrische Störungen, periphere Neuropathie, Parkinsonismus und Dysautonomie (Impotenz, Inkontinenz). Keines der genannten Merkmale ist syndromspezifisch. Das FXTAS überschneidet sich signifikant mit dem cerebellären Subtyp der Multiplen Systematrophie (MSA-C) und mit den spätmanifestierenden Kleinhirnataxien. Das alleinige obligate Merkmal des FXTAS ist ein unmethyliertes, transkriptionsaktives *FMR1*-Gen mit verlängertem CGG-Repeat.

Das Risiko von Männern, an FXTAS infolge einer *FMR1*-Prämutation zu erkranken ist altersabhängig. Es liegt bei 17 % (50–59 Jahre), 38 % (60–69 Jahre), 47 % (70–79 Jahre) und 75 % (≥80 Jahre) wobei der Tremor typischerweise vor der Ataxie auftritt. Bei Prämutationsträgerinnen tritt ein FXTAS viel seltener auf [2].

Bei Jungen mit einer Prämutation wird im Vergleich zur Allgemeinbevölkerung eine erhöhte Prävalenz von ADHS und Autismus-Spektrum-Störungen beschrieben [3].

### Fragile-X assoziierte prämature Ovarialinsuffizienz (FXPOI)

FXPOI ist das Auftreten von prämaturer Ovarialinsuffizienz bei Frauen, insbesondere auch bei familiären Fällen. Etwa 20 % aller Prämutationsträgerinnen entwickeln eine frühzeitige Ovarialinsuffizienz mit einer Menopause vor dem 40. Lebensjahr. Das Auftreten einer POI ist bei diesen Frauen damit etwa 20x höher als im Bevölkerungsdurchschnitt, das bei etwa 1 % liegt [4]. In ungefähr 14 % aller familiären Fälle und etwa 2,5 % der sporadischen Fälle kann eine Prämutation bei den betroffenen Frauen nachgewiesen werden. In der Regel zeigen die betroffenen Frauen einen hypergonadotropen Hypogonadismus mit den entsprechend biochemisch nachgewiesenen veränderten Hormonparametern in Form eines erhöhten FSH-Spiegels. Die pathophysiologische Ursache scheint nach heutigem Kenntnisstand ebenfalls eine Toxizität der überexprimierten *FMR1*-mRNA zu sein, wobei die zellulären Mechanismen noch unklar sind.

## Indikationen

Molekulargenetische Untersuchungen des *FMR1*-Gens werden veranlasst zur Differentialdiagnostik, Anlageträgerdiagnostik und zur Pränataldiagnostik des Fragilen-X-Syndroms sowie zur Differentialdiagnostik bei klinischem Verdacht auf FXTAS oder FXPOI. Die Anlageträgerdiagnostik in Familien mit Fragilem-X-Syndrom beinhaltet unvermeidbar einen prädiktiven FXTAS-Gentest. Entsprechend sind die Bestimmungen des Gendiagnostikgesetzes (GenDG) vor der Untersuchung zu beachten.

Beim **Fragilen-X-Syndrom** besteht eine Indikation zur Absicherung der klinischen Verdachtsdiagnose (**Differentialdiagnostik**) in der Regel bei allen Graden einer kognitiven Beeinträchtigung, unabhängig davon, ob die Familienvorgeschichte des (männlichen oder weiblichen) Patienten auf eine X-chromosomale Vererbung der Beeinträchtigung hinweist. Die Untersuchung ist auch begründet, wenn keine der weiteren, beim Fragilen-X-Syndrom häufigen Merkmale offensichtlich sind. Eine Indikation zur **Anlageträgerdiagnostik** ist bei allen potentiellen und vielen obligaten Überträgerinnen gegeben. Die Klärung, ob bei der Mutter eines betroffenen Kindes ein vollmutiertes oder prämutiertes *FMR1*-Gen oder bei der Tochter eines männlichen Überträgers ein prämutiertes Allel mit weniger oder mehr als 90 Tripletts vorliegt, ist von Bedeutung für die Einschätzung der Wahrscheinlichkeit, mit der das Fragile-X-Syndrom bei einem (weiteren) Kind auftritt (Tabelle 2). Bei klinisch unauffälligen Jungen und Männern hat ein Gentest zur Klärung der Anlageträgerschaft diesbezüglich keine Relevanz. Die **pränatale Diagnostik** ist in der Regel nur bei Schwangeren angezeigt, deren Überträgerschaft (Vollmutation oder Prämutation) nachgewiesen ist. Bei einer Prämutation des Vaters ist eine vorgeburtliche Diagnostik nicht indiziert, da Söhne grundsätzlich das mütterliche X-Chromosom erben. Bei der Weitergabe des prämutierten Allels vom Vater an die Töchter wird es nicht zur Vollmutation expandieren. Die Töchter können sich bei Erreichen der Einwilligungsfähigkeit auf eine Anlageträgerschaft testen lassen.

Eine Indikation zur differentialdiagnostischen Untersuchung des *FMR1*-Gens zum Nachweis oder Ausschluss eines prämutierten CGG-Repeats besteht auch bei allen Männern und Frauen mit klinischen Zeichen eines **FXTAS**, insbesondere Intensionstremor, Parkinsonismus und/oder Gangataxie. Ein *FMR1*-Prämutationstest ist ferner angezeigt bei Frauen mit vorzeitiger Ovarialinsuffizienz (**POI**) oder auffälliger Familienanamnese einer POI. Erhöhte FSH-Spiegel und Menstruationsstörungen bei Frauen unter 40 Jahren sind Zeichen einer beginnenden POI und somit eine Indikation zur Testung auf FXPOI.

## Molekulargenetische Ursachen

Die bei weitem häufigste **genetische Ursache** des Fragilen-X-Syndroms ist ein **inaktiviertes, aberrant methyliertes*****FMR1*****-Gen**, dessen CGG-Repeat über 200 Tripletts lang ist („**Vollmutation**“). Die Repeatexpansion allein führt nicht zum Fragilen-X-Syndrom. Jedoch wird die DNA aufgrund der Länge des CGG-Repeats und des damit hohen GC-Gehalts aberrant gefaltet und dadurch in der Regel methyliert. Hierbei handelt es sich um eine aberrante, von der Repeatexpansion provozierte DNA-Modifikation. Die Methylierung erstreckt sich über den Promotor hinaus auf einen großen Abschnitt des X-Chromosoms und ist mit einer Umstrukturierung des Chromatins verbunden. Durch die Methylierung ist die Transkription des *FMR1*-Gens mehr oder weniger komplett inhibiert. Die Repeatexpansion (Mutation) und die Hypermethylierung (epigenetische Modifikation) sind räumlich und zeitlich voneinander getrennte Ereignisse (s. u.). Die **pathophysiologische** Ursache des Fragilen-X-Syndroms ist ein Verlust des funktionellen Genproduktes (FMR1-Protein, **FMRP**). Dieser kann auch durch andere *FMR1*-Mutationen verursacht sein. FMRP reguliert die Translation postsynaptischer Proteine in Nervenzellen mit Glutamat-Rezeptoren und fördert den Aufbau stabiler neuronaler Netzwerke.

**Tab. 1:** Genotyp-Phänotyp Beziehungen.

Genotyp^*^	Bezeichnung	Phänotyp
5 bis 44	Normalbereich	Normalpersonen
45 bis 54	Intermediärbereich (Grauzone)	Normalpersonen
55 bis ∼200	Prämutationsbereich	Normale männliche und weibliche Überträger des Fragilen-X-Syndroms. Männliche und weibliche FXTAS-Patienten. Etwa 10 % aller Frauen mit primärer Ovarialinsuffizienz
ab ∼200	Vollmutationsbereich	Männliche und weibliche Patienten mit Fragilem-X Syndrom^**^. Weibliche und männliche Überträger des Fragilen-X-Syndroms^***^

^*^Triplettzahl.

^**^Alle Männer und etwa 60 % aller Frauen mit *aberrant* methyliertem inaktiviertem *FMR1*-Gen.

^***^Alle Frauen mit voll expandiertem, aber nur auf dem inaktiven X Chromosom methylierten *FMR1*-Gen (*normale* Methylierung). Etwa 40 % aller Frauen mit voll expandiertem und *aberrant* methyliertem *FMR1*-Gen (Methylierung auf dem aktiven X-Chromosom). Alle Männer mit voll expandiertem aber unmethyliertem *FMR1*-Gen.

Das vollmutierte *FMR1*-Gen kommt bei betroffenen männlichen Patienten und bei einem Teil der weiblichen Überträger vor (**Tabelle****1**). Es wird stets von der Mutter ererbt. Diese kann selbst Trägerin eines vollmutierten *FMR1*-Gens sein. Meistens trägt sie jedoch ein Allel, dessen CGG-Repeat nur 55 bis ∼200 Tripletts aufweist. Eine Repeatverlängerung dieser Größenordnung nennt man **Prämutation**. Der Begriff bezeichnet eine Veränderung des CGG-Repeats, die keine Auswirkung auf die neuronale Entwicklung des Trägers hat. Solche *FMR1*-Allele werden transkriptional überexprimiert. Sie können zu neurodegenerativen Veränderungen führen und ein **FXTAS** hervorrufen (s. u.). Frauen mit prämutiertem *FMR1*-Allel haben etwa zwanzigmal häufiger als andere Frauen eine primäre Ovarialinsuffizienz (**POI**). Prämutierte CGG-Repeats können eine Vorstufe der Vollmutation sein, die zum Fragilen-X-Syndrom führt (**Tabelle****2**).

**Tab. 2:** Risiko einer Vollmutation bei Kindern von Überträgerinnen (Daten aus Nolin et al. [5]).

Länge des maternalen Repeats	Risiko (%)	Kinder mit Vollmutation/alle Kinder
45–49	0	0/98
50–54	0	0/102
55–59	0,5	1/197
60–64	1,7	2/115
65–69	7	6/85
70–74	21	18/84
75–79	47	47/99
80–84	62	60/96
85–90	81	34/42

Viele Trägerinnen eines prämutierten *FMR1*-Allels erben dieses nicht von der Mutter, sondern von ihrem Vater. Ein männlicher Überträger vererbt sein (prämutiertes) *FMR1*-Gen an jede Tochter, allerdings nie an einen chromosomal unauffälligen Sohn, da das *FMR1*-Gen auf dem X-Chromosom liegt. Anders als bei den Kindern von Überträgerinnen tritt die Vollmutation bei den Töchtern männlicher Überträger nicht auf. Sogar die Töchter von Männern mit vollmutiertem *FMR1*-Gen ererben nur ein prämutiertes Allel. Dies weist darauf hin, dass das FMRP im Rahmen der Spermatogenese benötigt wird und dass Vollmutationen bei Männern nur somatisch vorkommen. Die aberrante epigenetische Modifikation und die Inaktivierung des vollmutierten *FMR1*-Gens erfolgt erst nach dessen Weitergabe an die nächste Generation, und zwar in den ersten embryonalen Entwicklungswochen (1. Trimenon). Ein maternal oder paternal ererbtes prämutiertes Allel kann aufgrund der mitotischen Instabilität hinreichend langer unmethylierter CGG-Repeats auch postzygotisch bis in den Vollmutationsbereich expandieren. Allerdings kommt es dann offensichtlich nicht mehr zu einer aberranten Methylierung.

Die Bezeichnung „Prämutation“ besagt nicht, dass das verlängerte Repeat zu irgendeinem zukünftigen Zeitpunkt weiter expandieren wird. Ob ein bestimmtes Allel aus dem Prämutationsbereich zur Vollmutation expandieren kann, lässt sich erst dann sicher sagen, wenn es bereits an einen betroffenen Nachkommen weitergegeben wurde. Das kürzeste beobachtete Prämutationsallel, das bei der Weitergabe an ein Kind vollständig mutierte, hatte 56 CGGs [6]. Die Wahrscheinlichkeit, dass eine Überträgerin ein prämutiertes Allel als Vollmutation weitergibt, hängt von der Länge des prämutierten Repeats ab (**Tabelle****2**). Auch bei einer mütterlichen Triplettzahl zwischen 100–200 und selbst bei Überträgerinnen mit vollmutiertem *FMR1*-Allel kann ein Kind ausnahmsweise entgegen der Erwartung kein vollmutiertes sondern ein prämutiertes Allel ererben und frei von Symptomen des Fragilen-X-Syndroms bleiben.

CGG-Repeats mit 45 bis 54 Tripletts werden als **Grauzonenallel** (oder „intermediäres“ Allel) bezeichnet (**Tabelle****1**). Bei einem Grauzonenallel ist eine Expansion zur Vollmutation in der unmittelbar folgenden Generation noch nie beobachtet worden und demzufolge sehr unwahrscheinlich. Grauzone bedeutet, dass diese Repeats stabil oder instabil vererbt werden können. Etwa 10 % der Allele mit 45–49 CGG-Repeats werden instabil an die Folgegeneration weitergegeben, wobei eine Verlängerung von maximal mit 1–3 Tripletts zu erwarten ist. Bei Allelen mit 50–54 CGG-Repeats zeigt etwa jedes vierte eine Instabilität und einige davon expandieren in der Folgegeneration zur Prämutation. Die mitotische Stabilität eines CGG-Repeats wird auch durch die Anzahl und Position von AGG-Unterbrechungen bestimmt, die beim Gentest ermittelt werden kann, um das potentielle Verhalten eines Grauzonenallels besser prognostizieren zu können. Dies findet allerdings in der routinemäßigen Diagnostik in der Regel keine Anwendung.

## Anforderungen des Labors

Die grundlegenden Anforderungen, die bei jeder molekulargenetischen Untersuchung im Bereich der Humangenetik zu erfüllen sind, regelt insbesondere das Gendiagnostikgesetz, sowie die Richtlinie der Bundesärztekammer (RiliBÄK) und ggf. die Akkreditierungsnorm EN ISO15189. Zu den spezifischen fachkundlichen Voraussetzungen zur Durchführung molekulargenetischer Untersuchungen des *FMR1*-Gens gehört die Kenntnis aller echten und artifiziellen Resultate, die bei der Untersuchung unauffälliger und betroffener männlicher und weiblicher Individuen auftreten können. Der (fachlich qualifizierte) Laborleiter muss mit dem Phänomen der X-Inaktivierung, der damit verbundenen Methylierung des *FMR1*-Promotors vertraut sein und die Methylierungsmuster kennen, die in untersuchten Zellen (Leukozyten, Chorionzellen, Fruchtwasserzellen) im Normalfall vorliegen. Er muss zwischen der normalen *FMR1*-Methylierung auf inaktiven X-Chromosomen und der aberranten Methylierung vollmutierter Allele unterscheiden können und ihm sollten die bei Trägerinnen eines prämutierten oder vollmutierten *FMR1*-Gens zu beobachteten Abweichungen von einer zufälligen X-Inaktivierung vertraut sein.

### Methoden zum Nachweis normaler und expandierter FMR1-Allele

Ein zuverlässiges Verfahren zum direkten Nachweis und zur Charakterisierung expandierter CGG-Repeats als „unauffällig“, „prämutiert“ oder „vollmutiert“ ist der genomische Southern-Blot. Mittlerweile werden aber auch sehr häufig effiziente PCR-basierte Nachweisverfahren angewendet. Einige dieser Tests erfassen neben normalen auch prämutierte und vollmutierte FMR1-Allele. Allerdings sind die Ergebnisse bei Expansionen, die in der Regel als somatisches Mosaik aus Zellen mit unterschiedlich stark expandierten oder kontrahierten CGG-Repeats bestehen, häufig nicht deckungsgleich mit den Ergebnissen aus Southern-Blot-Analysen. Dies kann zu falsch-negativen Interpretationen führen, besonders dann, wenn auch Zellen mit einem normal großen Repeat enthalten sind, wobei dieses dann bevorzugt oder ausschließlich amplifiziert wird. Dies sollte bei der PCR-basierten Diagnostik beachtet werden.

#### Genomischer Southern-Blot

Die für den Southern-Blot benötigte Menge an hochmolekularer genomischer DNA wird in der Regel aus 2 bis 5 ml EDTA-Blut gewonnen. Die Methode der DNA-Extraktion beeinflusst die Qualität der DNA, so dass säulenbasierte Methoden sich häufig nicht dazu eignen. Der Southern-Blot ermöglicht den zuverlässigen Nachweis aller normalen, prämutierten und vollmutierten CGG-Repeats sowie eine Bestimmung der Methylierung des *FMR1*-Promotors. Das seit 1990 zur direkten Genotypdiagnostik beim Fragilen-X-Syndrom eingesetzte und vielfach validierte Verfahren stellt weiterhin den „goldenen Standard“ dar. Zum Nachweis oder Ausschluss einer Expansion des CGG-Repeats werden pro Test etwa 10 µg intakte hochmolekulare **genomische** DNA komplett mit Restriktionsenzymen gespalten, wobei neben Einzelspaltungen mit *Eco*RI, *Hind*III oder *Pst*I häufig Doppelspaltungen mit *Eco*RI oder *Hind*III plus einem methylierungssensitivem Enzym (*Eag*I, *Bss*HII, *Nru*I) durchgeführt werden. Die Auftrennung der Fragmente erfolgt in Agarosegelen in Abwesenheit von Ethidiumbromid, da EtBr als interkalierender Farbstoff, speziell in dem GC-reichen Repeat, die Laufeigenschaften der Moleküle im Gel verändert. Die Restriktionsfragmente des *FMR1*-Gens mit dem CGG-Repeat werden durch Hybridisierung mit spezifischen Sonden (z. B. StB12.31Ox1.9, Ox0.55 und Ox0.48 sind äquivalent zu StB12.3 bei EcoRI und HindIII-Spaltung. oder Ox0.552Ox0.48 ist äquivalent zu Ox0.55 bei PstI-Spaltung. oder anderen äquivalenten Sonden) detektiert. Bei methylierungssensitiver Restriktion kann bei weiblichen Personen das aktive und inaktive X-Chromosom getrennt dargestellt werden. Bei der Darstellung beider Normalfragmente beweist das keineswegs, dass zwei verschiedene Normalallele vorliegen und kein expandiertes Allel, sondern lediglich, dass zumindest ein normales Allel in methylierter (inaktiver) und unmethylierter (aktiver) Form vorkommt. Für den Nachweis kleiner prämutierter und intermediärer Allele werden am besten Restriktionsenzyme gewählt, die kleinere Fragmente ergeben (z. B. *Pst*I), weil hier die Auflösung besser ist als 30 bp (entsprechend 10 Tripletts). Hier kann die PCR-Analyse bis auf ein Triplett genaue Ergebnisse liefern. Vollmutierte *FMR1*-Allele liegen meistens nicht als Einzelbande, sondern als ein Muster mehrerer Banden (**somatisches Mosaik**) vor und sind fast immer methyliert. Die Methylierung betrifft alle oder nur einen Anteil der Fragmente (**Methylierungsmosaik**). Häufiger besteht ein **Genotypmosaik** mit prämutiertem und vollmutiertem *FMR1*-Allel. Bei manchen phänotypisch unauffälligen oder mit FXTAS betroffenen männlichen Überträgern/Patienten finden sich überwiegend oder ausschließlich unmethylierte vollmutierte *FMR1*-Genfragmente, deren Hybridisierungssignal als breiter Schmier ausgeprägt ist, sich manchmal über den gesamten Bereich der Repeatlängen ausdehnt und bei erhöhtem Hybridisierungshintergrund nur schwer erkannt wird. Diese Männer können FXTAS entwickeln, zeigen aber keine klassischen Merkmale eines FXS und werden als *high functioning males* bezeichnet. Bei bis zu 1 % aller männlichen Patienten detektieren die diagnostischen Sonden kein Restriktionsfragment, weil dem *FMR1*-Gen der entsprechende Abschnitt fehlt. In etwa 1–2 % der somatischen Mosaike findet sich neben mehreren expandierten Fragmenten auch ein Fragment normaler Größe. Bei inkompletter Spaltung, die unbedingt zu vermeiden ist, treten meistens sogenannte **Geisterbanden** auf, deren Muster im Gegensatz zu den Banden expandierter CGG-Repeats typisch ist für das jeweilige Restriktionsenzym.

#### PCR-Analyse des CGG-Repeats mit flankierenden Primern (CGG-PCR)

Eine zuverlässige Amplifikation des CGG-Repeats aus genomischer DNA erfordert eine leistungsfähige DNA-Polymerase und ein PCR-Protokoll, das die äußerst CG-reichen DNA-Moleküle effizient denaturieren und in allen Zyklen einzelsträngig halten kann.

Seit ein paar Jahren werden kommerzielle PCR-Kits angeboten, mit denen prämutierte und vollmutierte CGG-Repeats amplifiziert und zuverlässig nachgewiesen werden können. Probleme können hierbei insbesondere bei sehr heterogenen Mosaiken auftreten, da kürzere PCR-Fragmente besser amplifiziert werden.

In einigen Laboren werden auch in-house PCR-Protokolle zur Amplifikation des CGG-Repeats angewendet. Mit diesen gelingt teilweise die Amplifikation von Genfragmenten mit mehr als 100 CGG-Wiederholungen und ihre Darstellung durch Sequenzgelelektrophorese unter Verwendung fluoreszenzmarkierter Primer. Nach Auswertung von Ringversuchen ist offensichtlich, dass die Repeatlänge expandierter Allele nicht zuverlässig auf ein Triplett genau bestimmt werden kann. Die Ergebnisse verschiedener Laboratorien weichen bis zu 10 % vom Referenzwert ab. Die elektrophoretische Mobilität von DNA-Fragmenten mit einer CGG-Repeatsequenz unterscheidet sich deutlich von der herkömmlicher „size standards“ gleicher Länge. Deshalb sollen bei der CGG-PCR zusätzlich zur Test-DNA in erster Linie *FMR1*-Allele mit exakt bekannter Triplettzahl (z. B. 30, 40 und 50 CGGs) ko-amplifiziert und mit aufgetrennt werden, die als Größenstandards dienen.

#### PCR durch das CGG-Repeat mit drei Primern (Triple-PCR, T-PCR)

Bereits 1996 wurde von Warner et al. [7] eine Triple-PCR (**T-PCR**) Methode beschrieben, mit deren Hilfe expandierte CAG-Repeat Sequenzen detektiert werden können, die für eine herkömmliche PCR-Amplifikation zu lang sind. Modifikationen der T-PCR werden auch bei der Untersuchung des *FMR1*-Gens angewandt, insbesondere wenn ein Blot zu aufwendig ist oder nur eine minimale DNA-Menge zur Verfügung steht. Die T-PCR kann zwar die Existenz eines expandierten, mit flankierenden Primern nicht amplifizierbaren CGG-Repeats aufzeigen, eine Klassifizierung nach der Repeatlänge (Prämutation oder Vollmutation) ist aber in der Regel nicht möglich.

#### MS-PCR zum Nachweis methylierter FMR1-Allele

Methylierte Allele können durch eine Standard-PCR nicht von unmethylierten Allelen unterschieden werden. Es besteht jedoch die Möglichkeit durch Spaltung der genomischen DNA mit methylierungssensitivem (MS) Restriktionsenzym und anschließender PCR den Methylierungsstatus zu ermitteln. Dieses Verfahren wird inzwischen auch von kommerziellen Diagnostika-Firmen angeboten. Dazu wird die DNA mit einem methylierungsunabhängigen Restriktionsenzym und parallel dazu mit einem methylierungssensitiven Restriktionsenzym geschnitten. Beide DNA werden in separater PCR mit verschiedenfarbigen fluoreszenzmarkierten Primern amplifiziert, detektiert und quantifiziert.

## Vorgehensweisen bei der Differentialdiagnostik des Fragilen-X-Syndroms

Die differentialdiagnostische Fragestellung impliziert den Auftrag zum Nachweis oder Ausschluss der *FMR1*-Vollmutation bei einem männlichen oder weiblichen Patienten zur Überprüfung einer klinischen Verdachtsdiagnose. Alle hierfür notwendigen Laborergebnisse können einem genomischen Southern-Blot entnommen werden. Empfohlen wird eine Doppelspaltung mit methylierungssensitivem Enzym (z. B. *Eco*RI plus *Eag*I oder *Eco*RI plus *Nru*I) oder zwei Einzelspaltungen (z. B. *Eco*RI oder *Hind*III und *Pst*I). Seit ein paar Jahren werden häufig auch effiziente PCR-basierte Nachweisverfahren angewendet, die in der Lage sind, neben normalen auch prämutierte und vollmutierte FMR1-Allele sicher zu erfassen

Wurde bei einem betroffenen männlichen oder weiblichen Probanden eine Vollmutation nachgewiesen, ist die Diagnose eines Fragilen-X-Syndroms definitiv gesichert. Entsprechendes gilt, wenn die Sonde bei einem Jungen kein *FMR1*-Genfragment detektiert. Wurde bei einem Jungen nur ein normal großes Fragment im Blot detektiert, ist nicht nur eine Vollmutation sondern auch eine Deletion des *FMR1*-Promotors ausgeschlossen, denn alle diagnostischen *FMR1*-Gensonden detektieren Fragmente, die auch den Promotor und die Startstelle der Transkription enthalten. Eine solche Deletion ist die genetische Ursache bei bis zu 1 % aller Fälle eines Fragilen-X-Syndroms. Bei Frauen und Mädchen kann eine Deletion in der Promotorregion oft bei Doppelspaltung mit einem methylierungssensitiven Enzym erkannt werden, weil nur selten genau die Hälfte aller normalen X-Chromosomen inaktiviert ist. Ein mittels Southern-Blot erhobenes normales Untersuchungsergebnis schließt ein Fragiles-X-Syndrom nicht aus, weil selten auftretende Punktmutationen im *FMR1*-Gen (weniger als 0,1 % aller Fälle) durch diese Methode nicht entdeckt werden. Auch sehr seltene Deletionen und Duplikationen, die außerhalb der Restriktionsfragmente gelegen sein können, werden nicht detektiert. Diese können aber in einigen Fällen mit (methylierungssensitiver) MLPA (multiplex ligation-dependent probe amplification) dargestellt werden. Wir gehen davon aus, dass in hochwertigen Southern-Blots, die frei sind von breit geschmierten Hintergrundsignalen, alle diagnostisch relevanten Genotyp-Mosaike entdeckt werden.

Eine andere Vorgehensweise beginnt mit einem PCR-Screening. Das Resultat wird als „unauffällig“ befundet, wenn (a) bei einem männlichen Patienten ein normales CGG-Repeat detektiert wurde und (b) bei einer weiblichen Patientin zwei normale CGG-Repeats verschiedener Länge nachgewiesen wurden. Ein Fragiles-X-Syndrom ist aber nicht ausgeschlossen, weil – wie auch beim Southern-Blot – nicht alle kausalen *FMR1*-Genveränderungen detektiert werden. Ferner gibt es seltene diagnostisch relevante somatische Mosaike, bei denen die PCR ein normales Signal ergibt, wenn insgesamt nur wenige Moleküle eines normalen CGG-Repeats vorhanden sind. Wurde mit einer PCR-Methode, bei der vollmutierte Repeats im Allgemeinen nicht detektiert werden, bei einem betroffenen Jungen kein Signal erhalten, sollte ein abschließender Befund erst dann erstellt werden, wenn das vermutete vollmutierte Allel oder dessen aberrante Methylierung explizit nachgewiesen wurde. Kann dieser Nachweis nicht erbracht werden, muss im Befundbrief auf die diagnostische Unsicherheit dieses Ergebnisses hingewiesen werden. Wurde bei einem Mädchen nur ein PCR-Signal erhalten, das von einem oder zwei gleich langen Repeats stammen kann, muss ebenfalls ein adäquater Test zum Nachweis oder Ausschluss eines vollmutierten Allels erfolgen.

## Vorgehensweisen bei der Anlageträgerdiagnostik des Fragilen-X-Syndroms

Bei einer Ratsuchenden ohne klinische Zeichen eines Fragilen-X-Syndroms soll der Nachweis oder Ausschluss eines prämutierten oder vollmutierten *FMR1*-Allels erfolgen. Die Indikation für diese Untersuchung kann eine familiäre geistige Behinderung sein, ein bekanntes Fragiles-X-Syndrom in der Familie, eine vorzeitige Ovarialinsuffizienz (POI) bei ihr oder in ihrer Familie oder ein FXTAS in der Familie sein.

Die diagnostische Vorgehensweise einschließlich der für die Ergebnisinterpretation geltenden Einschränkungen entspricht der bei differentialdiagnostischen Fragestellungen. Im Southern-Blot wird ein prämutiertes Fragment nach *Eco*RI- oder *Hind*III-Einzelspaltung jedoch nicht hinreichend zuverlässig von einem Normalfragment unterschieden und nachgewiesen. Deshalb ist zusätzlich zur genaueren Abklärung der Länge des prämutierten CGG-Repeats eine PCR-Analyse und / oder ein *Pst*I-Blot zu empfehlen.

Im Bereich zwischen 55 und 90 Tripletts steigt mit zunehmender Repeatlänge das Risiko einer Ratsuchenden, ein Kind mit vollmutiertem Allel zu bekommen (**Tabelle****2**). Die meisten Prämutationen liegen in diesem Längenintervall und sind durch stabile PCR-Techniken oft direkt nachweisbar.

Prämutierte Allele mit mehr als 130 Tripletts zeigen immer eine mehr oder weniger ausgeprägte mitotische Instabilität und erzeugen postzygotisch ein somatisches Mosaik mit sowohl größeren Allelen, deren Länge im Vollmutationsbereich liegen kann, als auch kürzeren Repeats, deren Länge im Normalbereich liegen kann. Es ist zu beachten, dass postzygotisch in den Normalbereich verkürzte prämutierte Allele bei der PCR unter Umständen mit einem zweiten Normalallel verwechselt werden können. Im Zweifel sollte bei ungewöhnlichen Peakhöhen und ungewöhnlichen Repeatlängen ein klärender Southern-Blot erfolgen, da selbst leistungsstarke PCR-Analysen entsprechende Mosaike möglicherweise nicht sicher detektieren.

Der Befund eines prämutierten oder vollmutierten Allels bei einer Überträgerin soll, falls aufgrund der individuellen Situation der Ratsuchenden nicht unangemessen, mit Informationen zur Pränataldiagnostik oder Präimplantationsdiagnostik des Fragilen-X-Syndroms ergänzt werden.

## Vorgehensweise bei FXTAS-Gendiagnostik

Die molekulare Ursache des FXTAS beschränkt sich nach derzeitigem Kenntnisstand auf Repeatexpansionen im Prämutationsbereich des *FMR1*-Gens, also auf 55 bis 200 CGG-Wiederholungen. Die diagnostische Fragestellung und die Vorgehensweise entspricht somit im Prinzip der einer Anlageträgerdiagnostik beim Fragilen-X-Syndrom. Der Auftrag beinhaltet aber keinen Test auf ein unmethyliertes CGG-Repeat mit der Länge eines vollmutierten Allels. Die untersuchten Personen sind meistens betroffene Männer und weniger häufig betroffene Frauen ab einen Alter von etwa 50 Jahren.

Wenn bei einem männlichen Probanden ein prämutiertes *FMR1*-Repeat gefunden wurde, gilt die klinische Diagnose eines FXTAS als gesichert. Über diese Feststellung hinaus muss bei der Ergebnisinterpretation festgestellt werden, dass der Patient ein Überträger des Fragilen-X-Syndroms ist, der ein prämutiertes CGG-Repeat an alle seine Töchter weitergibt, die somit obligate Überträgerinnen des Fragilen-X-Syndroms sind.

Abgesehen von der Anlageträgerdiagnostik in Familien mit Fragilem-X-Syndrom sind FXTAS-Gentests bei gesunden Personen eher selten. Bei einem positiven Untersuchungsresultat stehen derzeit nur für Männer prädiktive Informationen zur Verfügung, da die geringen Fallzahlen für Frauen noch keine sicheren Aussagen erlauben.

## Vorgehensweise bei der Pränataldiagnostik und Präimplantationsdiagnostik des Fragilen-X-Syndroms

Die Vollmutation des Fragilen-X-Syndroms ist pränatal diagnostizierbar an DNA aus Chorionzotten oder kultivierten bzw. nicht-kultivierten Fruchtwasserzellen bei männlichen und weiblichen Feten. Die Indikation ist gegeben, wenn bei der Mutter ein *FMR1*-Allel mit vollmutiertem oder prämutiertem CGG-Repeat bekannt ist. Neben dem diagnostischen Test zur Beantwortung der Fragestellung ist zusätzlich eine maternale Kontamination der fetalen DNA-Probe abzuklären. Ein Kontaminationstest ist mindestens dann erforderlich, wenn der Fet einen weiblichen Chromosomensatz hat und beide CGG-Repeats dem der beiden maternalen Allele größenmäßig entsprechen oder ein normales CGG-Repeat nachgewiesen wird, ohne dass das väterliche Allel bekannt ist und der diagnostische Test größere Expansionen nicht erfasst. Eine direkte Untersuchung des CGG-Repeats kann mit einem Southern-Blot und/oder einer PCR-Analyse erfolgen. Dabei ist es durchaus hilfreich mittels PCR-Analyse die Weitergabe des normalen mütterlichen *FMR1*-Allels an das erwartete Kind zu überprüfen. Bei weiblichem Fet ist auch die Untersuchung der DNA des Vaters sinnvoll, dessen CGG-Repeat die gleiche Länge haben kann wie das normale maternale Allel.

Das von den meisten Laboratorien zur molekulargenetischen Pränataldiagnostik bevorzugte Gewebe sind frisch präparierte Chorionzotten. In der Plazenta weiblicher Feten ist die X-Inaktivierung normaler *FMR1*-Gene – anders als in Leukozyten – nicht verknüpft mit einer Methylierung des Promotors. Jede Methylierung des *FMR1*-Gens in Chorionzotten ist aberrant. Ein möglicher Nachteil von Chorionzotten ist der relativ späte Zeitpunkt der aberranten Methylierung eines vollmutierten *FMR1*-Gens, wodurch Interpretationsprobleme bei Anwendung eines methylierungssensitiven Tests entstehen können. Gleichwohl ist die Befundinterpretation in dem nicht häufigen Fall eines unmethylierten vollmutierten *FMR1*-Gens, das im Prinzip mit einer normalen intellektuellen Entwicklung vereinbar ist, schwierig und sollte nicht ohne eine Überprüfung des Methylierungsstatus nach Amniozentese erfolgen. Die pränatale Diagnostik des Fragilen-X-Syndroms aus Fruchtwasserzellen hat den Nachteil, dass ohne Kultivierung die geringe fetale DNA-Menge in der Regel nicht ausreichend für eine Southern-Blot-Analyse ist und dass durch die Kultivierungszeit die Schwangerschaft in den meisten Fällen bereits weit fortgeschritten ist. Die aus der Amniozytenkultur gewonnene fetale DNA ist aufgrund des relativ hohen Anteils abgestorbener Amniozyten immer in hohem Maße degradiert, so dass für den Blot deutlich mehr DNA eingesetzt werden muss, um vergleichbare klare spezifische Hybridisierungssignale zu erhalten. Zum Ausschluss einer maternalen Kontamination des fetalen Untersuchungsmaterials ist eine Analyse polymorpher Markern obligater Bestandteil der Untersuchung.

Die Präimplantationsdiagnostik (PID) ist seit einigen Jahren in Deutschland unter bestimmten gesetzlich festgelegten Voraussetzungen möglich. Für das Fragile-X-Syndrom sind diese Bestimmungen anwendbar, so dass diese Untersuchung in spezialisierten Zentren möglich ist. Im Wesentlichen beruht die Diagnostik der PID derzeit auf einer indirekten Genotypanalyse, d. h. die verantwortliche Expansion des CGG-Repeats wird in der Regel nicht direkt bestimmt. Dazu werden Repeat-flankierende Marker untersucht, die in enger Kopplung mit dem CGG-Repeat stehen. Die Untersuchung erfolgt an genetischem Material aus Zellen von Blastomeren. Ein Sonderfall der PID ist die Polkörperdiagnostik, kurz PKD, wo das Untersuchungsmaterial aus Polkörpern gewonnen wird und die Analyse bereits vor der Befruchtung erfolgt.

Das FXS kann derzeit noch nicht durch einen nicht invasiven pränatalen Test (NIPT) detektiert werden.

## Befund

Ein Befundbericht mit wissenschaftlich fundierter humangenetischer Interpretation (Epikrise) ist wesentlicher Bestandteil der molekulargenetischen Diagnostik. Der Bericht soll klar, zielgerichtet, prägnant und akkurat sein. Er muss eine vollständige Befundinterpretation enthalten, die an der diagnostischen Fragestellung des Einzelfalles orientiert ist. Bei allen *FMR1*-assoziierten Syndromen sind die entsprechenden Hintergrundinformationen notwendig. Sie müssen korrekt und aktuell sein, sollen sich auf das zum Verständnis des Berichtes erforderliche Maß beschränken und nicht im Vordergrund des Berichtes stehen. Die insgesamt erforderlichen Inhalte eines Befundberichts sind in diversen nationalen und europäischen Leitlinien und Richtlinien (z. B. RiLiBÄK, S2k-Leitlinie AWMF 078-015 Humangenetische Diagnostik und genetische Beratung, Claustres et al. [8], Biancalana et al. [9]) vorgegeben.
